# Editorial: Machine Learning Approaches to Human Movement Analysis

**DOI:** 10.3389/fbioe.2020.638793

**Published:** 2021-01-22

**Authors:** Matteo Zago, Ana Francisca Rozin Kleiner, Peter Andreas Federolf

**Affiliations:** ^1^Dipartimento di Elettronica, Informazione e Bioingegneria, Politecnico di Milano, Milan, Italy; ^2^MOVA4all, São Paulo, Brazil; ^3^Department of Sport Science, University of Innsbruck, Innsbruck, Austria

**Keywords:** artificial intelligence, biomechanics, motor control, gait analysis, neural networks, motion analysis, motion classification, PCA

## Embracing Human Movement Complexity

Back in the mid-2000s, when Facebook was new and smartphones had still not become a major part of our everyday lives, researchers started to explore the use of Machine Learning in biomechanics. The road ahead appeared uncertain and people asked whether it was a new dawn or false hope? (Bartlett, 2006). Fifteen years later, we are in the middle of the era of data science, witnessing an unprecedented flourishing of techniques and applications. When large amounts of information can be collected and analyzed, the appeal and “unreasonable effectiveness of data” (Halevy et al., 2009) has found fertile ground in the study of complex biological and physical systems, human movement science among them.

The way we humans move, and the underlying cognitive control involved in this process is inherently complex, dynamic, multidimensional, and highly non-linear (Phinyomark et al., 2018). Machine Learning approaches enable us to embrace this complexity, working on three complementary tasks: predictive modeling, classification, and dimensionality reduction. With contributions from the five continents, the collection of papers in this Research Topic represents insightful viewpoints on the current landscape and potential new trends on the horizon.

## Estimation of Kinetics and Kinematics From Wearable Sensors

A model is a summary of the best knowledge of a system at the time it is investigated, capturing essential aspects that are critical in answering the question at hand. Predicting modeling maps input data to a given output and can be used to anticipate future events with confidence. It is not surprising that the availability of large datasets obtained from wearable sensors has promoted considerable advancements in the estimation of quantities that traditionally required expensive laboratory setups, such as ground reaction forces and derived variables. This is one of the main technological trends in recent research. The study by Mundt et al. discusses motion capture systems and how they retrieve kinetic (and kinematics) data with less expert knowledge and without expensive equipment. As they state, motion capture systems are going to “increase the availability of motion analysis to a wider range of people.” In other words, access to such systems enables a move toward pervasive healthcare systems (Zhou et al., 2020). Exploiting the power and versatility of artificial (deep) neural networks as universal function approximators, the contributions by Stetter et al. and Derie et al. estimated external knee moments and vertical loading during various locomotion tasks, including running and using a minimal set of IMUs. Dorschky et al. worked on the same topic by augmenting a measured inertial sensor dataset with simulated data to demonstrate how to efficiently estimate sagittal plane angles, joint moments, and ground reaction forces. Complimentary work by De Brabandere et al. shows how the kinetics of the hip and knee can be estimated using smartphone embedded sensors.

## Artificial Intelligence on the Move

The automatic classification of athletic tasks based on motion data gathered in real-world conditions with inertial sensors is another expanding area of investigation, as evidenced in papers by Benson et al., Clouthier et al., and Preatoni et al. They use neural networks together with Support-Vector Machines and k-Nearest Neighbors algorithms. Pattern-recognition was also made possible by a combination of unsupervised Principal Component Analysis (PCA) and Gaussian Mixture Model or Linear Discriminant Analysis. Another contribution by Remedios et al. describes an alternative way to objectively identify movement phenotypes without the need to *a-priori* prescribe movement features. Ross et al. discriminate élite from novice athletes, devising inertial features that capture motion details related to competition level. The recognition of movement patterns was also a key focus in the study by Suda et al., which used ground reaction forces to recognize foot-ankle movement strategies in long-distance runners.

## Computer Vision

Computer Vision represents a parallel trend, involving a combination of deep neural networks and simpler classification algorithms. Guerra et al. propose an interesting application of automatic pose recognition (also used in Zago et al. for gait analysis) and classification to trigger an alarm in frail individuals. The study by Gregori et al. focused on rehabilitation by developing a deep-learning method to automatically evaluate grasping actions in people with upper limb prostheses. Background segmentation and shape classification allowed Monezi et al. to automatically detect the three-dimensional location of multiple players in a basketball court.

## A Step Forward

Gait analysis is undoubtedly a collector of the data revolution. Instrumented gait assessment is routinely used to evaluate an individual's quality of life, morbidity, and/or mortality. Here, data science is a powerful complement to traditional approaches when handling large, heterogeneous, and sometimes noisy data sets (Ferber et al., 2016). The integration of machine learning with biomechanics not only simplifies the assessment of several interdependent parameters (Khera and Kumar, 2020) but also provides the opportunity for automated and unbiased analysis (Arac, 2020). Rethwilm et al. gained insights on trunk lean control in patients with Cerebral Palsy, combining PCA to binary logistic regression. In their technical paper, Burdack et al. explained how data filtering and unsupervised data reduction impacted gait classification based on ground reaction force data. Principal Component analysis is also the framework of analysis explored by Promsri and Federolf, who crafted a methodological paper explaining how to gain information about the coordinative structure of complex whole-body movements during balancing. In addition, De Roeck et al. focus on lower limb kinematics during deep squatting and in the forward lunge, devising a statistical model that predicts lower limb kinetics therein.

Zaroug et al. propose a method to predict lower limb kinematic trajectories during walking using long short-term memory (LSTM) neural networks. LSTM was also combined with convolutional neural networks by Yu et al. to predict pre-impact fall for older people. Notably, this approach was also implemented in a microcontroller unit featuring a working device. The practical translation of these techniques constitutes a crucial step that has not yet been undertaken that requires further exploration in future studies.

## Clinical Applications

Many papers in the Research Topic propose research-grade applications, and the effective combination of technology and data science will be topical in the near future. The seed of this trend is already visible. For instance, statistical shape modeling supported by logistic regression has clinical applications in automating the identification of surgically-relevant landmarks, as demonstrated by Cerveri et al.. de Araújo et al. showed how hand resting tremors could be used in the diagnosis of Parkinson's Disease. For patients in a similar condition, Lebel et al. worked on the prediction of motor performance based on visible symptomatology. A crucial issue is discussed in the work by Chia et al., which developed a decision support system based on gait kinematics, anthropometric characteristics, and physical examination and trained a system to learn the recommendations formulated by clinicians.

## What's Next

While carefully avoiding falling into a simplistic (and potentially dangerous) “idolatry of data,” we believe that the road is paved for rapid and inevitable (re)volutions. Assisting human decisions is among the most impactful advancements that data science and human movement science together can provide to medicine in the next decade (Jones et al., 2018). First data science can benefit education by supporting junior clinicians and potentially, later on, assisting in diagnosis and prognosis. In this journey, data is a powerful ally, but there is a need for machine learning to provide transparency and justifications of predictions (Halilaj et al., 2018; Horst et al., 2019). A framework to interpret deep learning features and the “magic inside the black box” is essential and significant efforts are currently being made toward creating explainable artificial intelligence (Côté-Allard et al.). Furthermore, as anticipated by many experts (Ferber et al., 2016; Halilaj et al., 2018), a cultural shift toward data sharing is necessary to achieve the required general validity (and constant upgradability) that will bring these systems into clinical practice.

The constant growth of computational power and wearable sensor miniaturization will also open pathways to pervasive real-time applications, exploiting the wealth of data available “out in the wild,” from marker-less motion capture to exercise monitoring and training assistance. To date, artificial intelligence does not simply provide new tools to study human motion. Rather, the way we study human motion is evolving thanks to artificial intelligence. By “following the data” (Halevy et al., 2009), we are pursuing unexplored and fascinating avenues of knowledge.

## Author Contributions

MZ, AK, and PF contributed equally to the writing of this editorial. All authors contributed to the article and approved the submitted version.

## Conflict of Interest

The authors declare that the research was conducted in the absence of any commercial or financial relationships that could be construed as a potential conflict of interest.
